# [*N*-(1-Aza­nidyl-2,2,2-trichloro­ethyl­idene)-2,2,2-trichloro­ethanimidamide]­copper(II)

**DOI:** 10.1107/S1600536812036124

**Published:** 2012-08-25

**Authors:** Namig G. Shikhaliyev, Abel M. Maharramov, Vasily M. Muzalevskiy, Valentine G. Nenajdenko, Victor N. Khrustalev

**Affiliations:** a Baku State University, Z. Khalilov St 23, Baku AZ-1148, Azerbaijan; b Chemistry Department, M.V. Lomonosov Moscow State University, Leninskie gory 1/3, Moscow 119991, Russian Federation; c X-Ray Structural Centre, A.N. Nesmeyanov Institute of Organoelement Compounds, Russian Academy of Sciences, 28 Vavilov St B-334, Moscow 119991, Russian Federation

## Abstract

The title compound, [Cu(C_4_H_2_Cl_6_N_3_)_2_], was obtained by the reaction of CCl_3_CN with ammonia in presence of CuCl. The Cu^II^ atom is located about an inversion centre. The mol­ecule consists of three planar units (one central square CuN_4_ and two C_2_N_3_ fragments), adopting a staircase-like structure. The six-membered metallocycles have a sofa conformation with the Cu atom out of the plane of the 1,3,5-triaza­penta­dienyl ligands by 0.246 (5) Å. The *ipso*-C atoms of the CCl_3_ substituents are slightly out of the 1,3,5-triaza­penta­dienyl planes by 0.149 (6) and −0.106 (6) Å. The CCl_3_ groups of each 1,3,5-triaza­penta­dienyl ligand are practically in the energetic­ally favourable mutually eclipsed conformation. In the crystal, the mol­ecules are packed in stacks along the *a* axis. The mol­ecules in the stacks are held together by two additional axial Cu⋯Cl inter­actions of 3.354 (2) Å. Taking the axial Cu⋯Cl inter­actions into account, the Cu^II^ atom exhibits a distorted [4 + 2]-octa­hedral coordination environment. The stacks are bound to each other by weak inter­molecular attractive Cl⋯Cl [3.505 (2)–3.592 (3) Å] inter­actions.

## Related literature

For a catalytic olefination reaction, see: Shastin *et al.* (2001 ▶); Korotchenko *et al.* (2001 ▶); Nenajdenko *et al.* (2003 ▶, 2004*a* ▶,*b* ▶,*c* ▶, 2005 ▶, 2007 ▶). For related compounds, see: Boča *et al.* (1996 ▶); Kajiwara *et al.* (2002 ▶); Zhang *et al.* (2005 ▶); Igashira-Kamiyama *et al.* (2006 ▶); Zheng *et al.* (2007 ▶); Figiel *et al.* (2010 ▶).
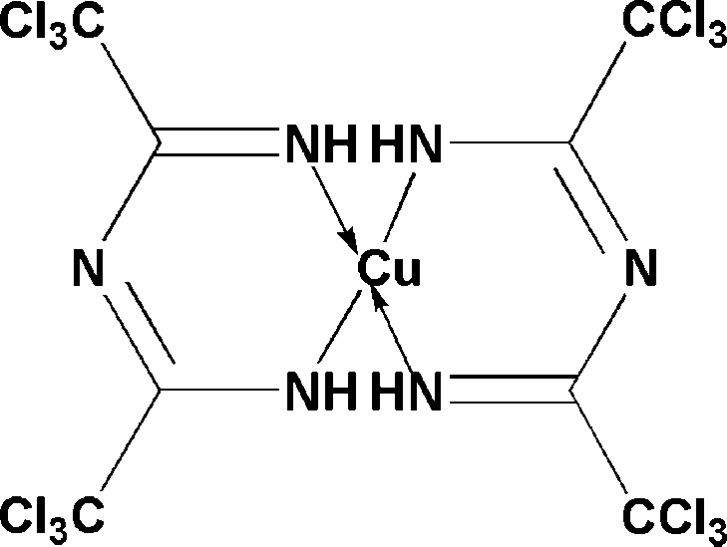


## Experimental

### 

#### Crystal data

[Cu(C_4_H_2_Cl_6_N_3_)_2_]
*M* *_r_* = 673.11
Triclinic, 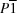

*a* = 5.9317 (17) Å
*b* = 9.078 (3) Å
*c* = 10.831 (3) Å
α = 98.475 (5)°
β = 97.525 (5)°
γ = 103.662 (5)°
*V* = 552.1 (3) Å^3^
*Z* = 1
Mo *K*α radiation
μ = 2.45 mm^−1^
*T* = 296 K
0.33 × 0.24 × 0.06 mm

#### Data collection

Bruker APEXII CCD diffractometer
Absorption correction: multi-scan (*SADABS*; Sheldrick, 2003 ▶) *T*_min_ = 0.499, *T*_max_ = 0.867
5662 measured reflections
2414 independent reflections
2108 reflections with *I* > 2σ(*I*)
*R*_int_ = 0.029

#### Refinement

*R*[*F* ^2^ > 2σ(*F* ^2^)] = 0.047
*wR*(*F* ^2^) = 0.133
*S* = 1.00
2414 reflections
124 parameters
H-atom parameters constrained
Δρ_max_ = 1.18 e Å^−3^
Δρ_min_ = −0.85 e Å^−3^

### 

Data collection: *APEX2* (Bruker, 2005 ▶); cell refinement: *SAINT* (Bruker, 2001 ▶); data reduction: *SAINT*; program(s) used to solve structure: *SHELXTL* (Sheldrick, 2008 ▶); program(s) used to refine structure: *SHELXTL*; molecular graphics: *SHELXTL*; software used to prepare material for publication: *SHELXTL*.

## Supplementary Material

Crystal structure: contains datablock(s) global, I. DOI: 10.1107/S1600536812036124/aa2069sup1.cif

Structure factors: contains datablock(s) I. DOI: 10.1107/S1600536812036124/aa2069Isup2.hkl

Additional supplementary materials:  crystallographic information; 3D view; checkCIF report

## Figures and Tables

**Fig. 1.**
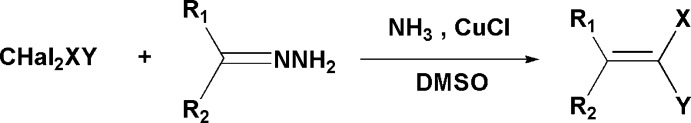
New catalytic olefination reaction as a general method for the preparation of alkenes; X and Y are H, Hal, CHal3, CN.

**Fig. 2.**
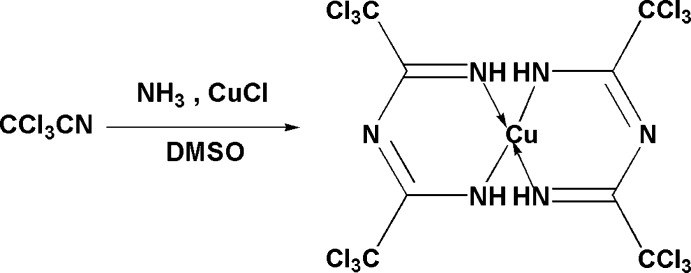
Reaction of CCl3CN with ammonia in presence of CuCl.

**Fig. 3.**
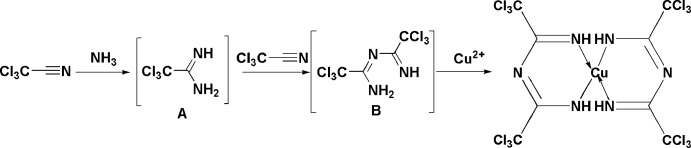
The stage-to-stage reaction mechanism of the formation of I.

**Fig. 4.**
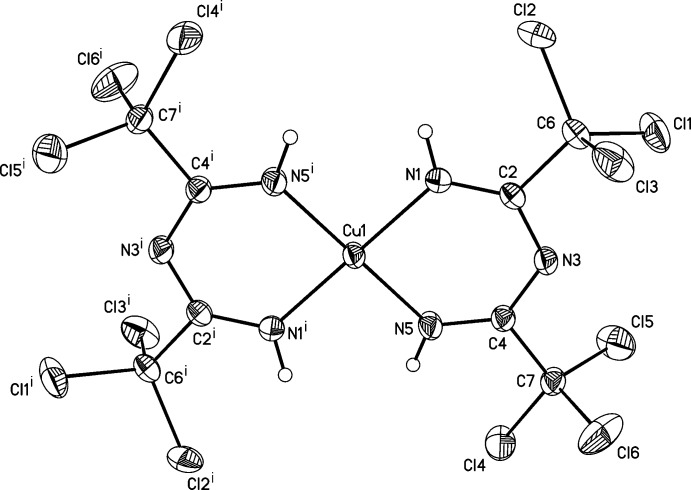
Molecular structure of I with the atom numbering scheme. Displacement ellipsoids are shown at the 50% probability level. H atoms are presented as small spheres of arbitrary radius. Symmetry code: (i) -x, -y + 2, -z + 2.

**Fig. 5.**
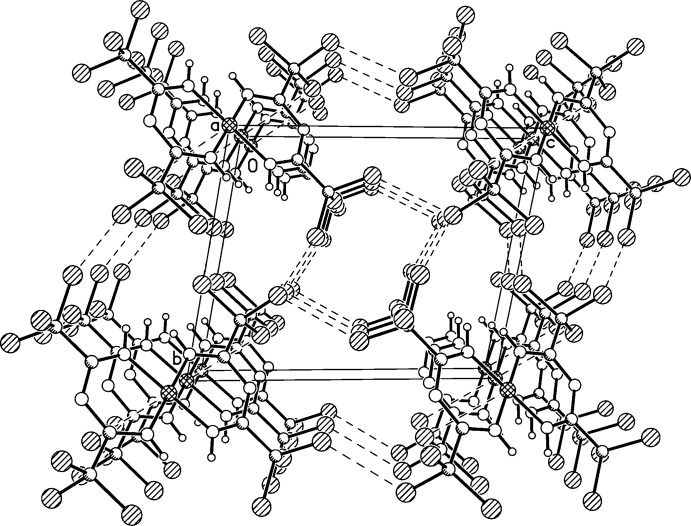
The crystal packing of I along the a axis. Dashed lines indicate the intermolecular axial Cu···Cl and attractive Cl···Cl interactions.

## supplementary crystallographic information

### Comment

Recently we have discovered a new catalytic olefination reaction as a general method for the preparation of alkenes from polyhalogenated compounds and hydrazones (Fig. 1) (Shastin *et al.*, 2001; Korotchenko *et al.*, 2001; Nenajdenko *et al.*, 2003, 2004*a*, 2004*b*, 2005, 2007).

During our study of the catalytic olefination reaction we have found that the reaction with trichloroacetonitrile demand the use of ethylenediamine as a base because in the case of ammonia no target alkene is formed (Nenajdenko *et al.*, 2004*c*). We decided to study the reaction of CCl_3_CN with ammonia in presence of CuCl more thoroughly and found that the formation of the title copper (II) chelate complex takes place (Fig. 2). The formation of this complex can be explained by high electrophilicity of trichloroacetonitrile (Fig. 3). At the first stage, ammonia reacts with CN bond to form amidine A as an intermediate. The subsequent reaction of A with second molecule of trichloroacetonitrile gives B. And finally, B reacts with CuCl_2_ resulting in the copper(II) complex I in a high yield. We believe that Cu^2+^is formed by oxidation of Cu^1+^ with CCl3CN as it was confirmed previously for catalytic olefination reaction.

The structure of the title compound I, C_8_H_4_N_6_Cl_12_Cu, was unambigouosly established by X-ray diffraction study (Fig. 4). The compound I crystallizes in the triclinic space group *P*-1 and there is a crystallographically imposed inversion centre at the Cu atom of each molecule. The Cu atom has a square-planar coordination. The 1,3,5-triazapentadienyl ligands are also planar (r.m.s. deviation is 0.021 Å). However, the six-membered metallocycles deviate significantly from the planarity and have a *sofa* conformation with the Cu atom out of the plane of the 1,3,5-triazapentadienyl ligands by 0.246 (5) Å. Thus, the molecule of I consists of the three planar units adopting the *staircase*-like structure. The similar molecular conformation has been previously observed in the related compounds (Zhang *et al.*, 2005; Igashira-Kamiyama *et al.*, 2006; Figiel *et al.*, 2010). Nevertheless, it is important to note that the analogous complexes can adopt the planar conformation also (Boča *et al.*, 1996; Kajiwara *et al.*, 2002; Zheng *et al.*, 2007). The *ipso*-C atoms of the CCl_3_-substituents are slightly out of the 1,3,5-triazapentadienyl planes by 0.149 (6) and -0.106 (6) Å. The CCl_3_-groups of each 1,3,5-triazapentadienyl ligand are practically in the energetically favorable eclipsed mutual conformation.

In the crystal, the molecules are packed in stacks along the *a* axis (Fig. 5). The molecules in the stacks are held together by the two additional axial Cu···Cl [Cu1···Cl1^i^ and Cu1···Cl1^ii^] interactions of 3.354 (2) Å. Taking the axial Cu···Cl interactions into account, the Cu atom attains the distorted [4 + 2]-octahedral coordination environment. The different stacks are bound to each other by weak intermolecular attractive interactions [Cu2···Cl2^iii^ 3.505 (2), Cu2···Cl2^iv^ 3.592 (3), Cu3···Cl4^v^ 3.516 (2) and Cu3···Cl6^vi^ 3.564 (2) Å] . Symmetry codes: (i) -*x*, -*y* + 2, -*z* + 2; (ii) *x* - 1, *y*, *z*; (iii) -*x* + 2, -*y* + 3, -*z* + 2; (iv) -*x* + 1, -*y* + 3, -*z* + 2; (v) *x* + 1, *y* + 1, *z*; (vi) -*x*, -*y* + 2, -*z* + 1.

### Experimental

A solution of trichloroacetonitrile (7.3 ml) in DMSO (15 ml) was dropped to a mixture of aqueous ammonia (5 ml) and freshly purified copper monochloride (0.3 g) during 3 min. upon keeping of the room temperature by the cooling on water-bath. The reaction mixture was stirred for 4 h. At the end of the reaction, the mixture was washed with water (150 ml) and filtered off. The formed product was re-crystallized from aqueous ethanol to give 1.47 g of red crystals of I. Yield is 73%. *M*.p. = 472–474 K.

### Refinement

The hydrogen atoms were placed in calculated positions with N–H = 0.86 Å and refined in the riding model with fixed isotropic displacement parameters [*U*_iso_(H) = 1.2*U*_eq_(N)].

### Crystal data

**Table:**

[Cu(C_4_H_2_Cl_6_N_3_)_2_]	*Z* = 1
*M**_r_* = 673.11	*F*(000) = 327
Triclinic, *P*1	*D*_x_ = 2.024 Mg m^−^^3^
Hall symbol: -P 1	Mo *K*α radiation, λ = 0.71073 Å
*a* = 5.9317 (17) Å	Cell parameters from 3874 reflections
*b* = 9.078 (3) Å	θ = 2.4–27.7°
*c* = 10.831 (3) Å	µ = 2.45 mm^−^^1^
α = 98.475 (5)°	*T* = 296 K
β = 97.525 (5)°	Plate, red
γ = 103.662 (5)°	0.33 × 0.24 × 0.06 mm
*V* = 552.1 (3) Å^3^	

### Data collection

**Table:**

Bruker APEXII CCD diffractometer	2414 independent reflections
Radiation source: fine-focus sealed tube	2108 reflections with *I* > 2σ(*I*)
Graphite monochromator	*R*_int_ = 0.029
φ and ω scans	θ_max_ = 27.0°, θ_min_ = 1.9°
Absorption correction: multi-scan (*SADABS*; Sheldrick, 2003)	*h* = −7→7
*T*_min_ = 0.499, *T*_max_ = 0.867	*k* = −11→11
5662 measured reflections	*l* = −13→13

### Refinement

**Table:**

Refinement on *F*^2^	Primary atom site location: structure-invariant direct methods
Least-squares matrix: full	Secondary atom site location: difference Fourier map
*R*[*F*^2^ > 2σ(*F*^2^)] = 0.047	Hydrogen site location: inferred from neighbouring sites
*wR*(*F*^2^) = 0.133	H-atom parameters constrained
*S* = 1.00	*w* = 1/[σ^2^(*F*_o_^2^) + (0.076*P*)^2^ + 0.84*P*] where *P* = (*F*_o_^2^ + 2*F*_c_^2^)/3
2414 reflections	(Δ/σ)_max_ < 0.001
124 parameters	Δρ_max_ = 1.18 e Å^−^^3^
0 restraints	Δρ_min_ = −0.85 e Å^−^^3^

### Special details

**Table:**

Geometry. All s.u.'s (except the s.u. in the dihedral angle between two l.s. planes) are estimated using the full covariance matrix. The cell s.u.'s are taken into account individually in the estimation of s.u.'s in distances, angles and torsion angles; correlations between s.u.'s in cell parameters are only used when they are defined by crystal symmetry. An approximate (isotropic) treatment of cell s.u.'s is used for estimating s.u.'s involving l.s. planes.
Refinement. Refinement of *F*^2^ against ALL reflections. The weighted *R*-factor *wR* and goodness of fit *S* are based on *F*^2^, conventional *R*-factors *R* are based on *F*, with *F* set to zero for negative *F*^2^. The threshold expression of *F*^2^ > σ(*F*^2^) is used only for calculating *R*-factors(gt) *etc*. and is not relevant to the choice of reflections for refinement. *R*-factors based on *F*^2^ are statistically about twice as large as those based on *F*, and *R*- factors based on ALL data will be even larger.

### Fractional atomic coordinates and isotropic or equivalent isotropic displacement parameters (Å^2^)

**Table:**

	*x*	*y*	*z*	*U*_iso_*/*U*_eq_	
Cu1	1.0000	1.0000	0.0000	0.03611 (19)	
Cl1	0.20230 (19)	0.81177 (15)	0.21612 (13)	0.0696 (3)	
Cl2	0.28874 (19)	0.61212 (13)	0.01081 (11)	0.0644 (3)	
Cl3	0.5633 (2)	0.65213 (14)	0.25606 (12)	0.0677 (3)	
Cl4	1.0465 (2)	1.42819 (12)	0.33037 (12)	0.0730 (4)	
Cl5	0.5879 (2)	1.27757 (17)	0.35949 (16)	0.0868 (5)	
Cl6	0.9881 (3)	1.18805 (17)	0.47247 (11)	0.0886 (5)	
N1	0.7097 (5)	0.8671 (3)	0.0275 (3)	0.0437 (7)	
H1	0.6365	0.7918	−0.0331	0.052*	
C2	0.6169 (5)	0.8813 (4)	0.1278 (3)	0.0358 (6)	
N3	0.6704 (5)	0.9993 (3)	0.2235 (3)	0.0441 (7)	
C4	0.8405 (6)	1.1234 (4)	0.2244 (3)	0.0353 (6)	
N5	0.9823 (6)	1.1456 (3)	0.1452 (3)	0.0446 (7)	
H5	1.0802	1.2351	0.1582	0.054*	
C6	0.4243 (6)	0.7471 (4)	0.1511 (4)	0.0421 (7)	
C7	0.8629 (7)	1.2489 (4)	0.3416 (3)	0.0425 (7)	

### Atomic displacement parameters (Å^2^)

**Table:**

	*U*^11^	*U*^22^	*U*^33^	*U*^12^	*U*^13^	*U*^23^
Cu1	0.0386 (3)	0.0301 (3)	0.0377 (3)	0.0011 (2)	0.0172 (2)	0.0035 (2)
Cl1	0.0495 (6)	0.0725 (7)	0.0932 (9)	0.0109 (5)	0.0390 (6)	0.0205 (6)
Cl2	0.0505 (5)	0.0531 (6)	0.0697 (7)	−0.0144 (4)	0.0029 (5)	0.0011 (5)
Cl3	0.0586 (6)	0.0634 (7)	0.0806 (8)	0.0026 (5)	0.0025 (5)	0.0422 (6)
Cl4	0.0951 (9)	0.0370 (5)	0.0731 (7)	−0.0102 (5)	0.0382 (6)	−0.0097 (5)
Cl5	0.0568 (7)	0.0793 (8)	0.1141 (11)	0.0171 (6)	0.0303 (7)	−0.0268 (8)
Cl6	0.1405 (14)	0.0778 (9)	0.0422 (6)	0.0311 (9)	−0.0047 (7)	0.0076 (5)
N1	0.0425 (15)	0.0386 (15)	0.0407 (15)	−0.0039 (12)	0.0135 (12)	−0.0039 (12)
C2	0.0343 (15)	0.0320 (15)	0.0399 (16)	0.0027 (12)	0.0094 (12)	0.0099 (12)
N3	0.0496 (16)	0.0379 (15)	0.0401 (15)	−0.0022 (12)	0.0199 (13)	0.0035 (12)
C4	0.0397 (16)	0.0312 (15)	0.0346 (15)	0.0058 (12)	0.0109 (12)	0.0063 (12)
N5	0.0515 (17)	0.0300 (14)	0.0478 (16)	−0.0025 (12)	0.0241 (13)	0.0003 (12)
C6	0.0341 (16)	0.0409 (17)	0.0505 (19)	0.0028 (13)	0.0118 (14)	0.0136 (14)
C7	0.0497 (19)	0.0364 (17)	0.0394 (17)	0.0066 (14)	0.0158 (14)	0.0014 (13)

### Geometric parameters (Å, º)

**Table:**

Cu1—N5	1.931 (3)	N1—C2	1.284 (4)
Cu1—N1	1.941 (3)	N1—H1	0.8600
Cl1—C6	1.749 (4)	C2—N3	1.322 (4)
Cl2—C6	1.767 (4)	C2—C6	1.537 (4)
Cl3—C6	1.759 (4)	N3—C4	1.321 (4)
Cl4—C7	1.762 (4)	C4—N5	1.282 (4)
Cl5—C7	1.742 (4)	C4—C7	1.544 (4)
Cl6—C7	1.736 (4)	N5—H5	0.8600
			
N5—Cu1—N1	87.83 (12)	Cu1—N5—H5	116.4
C2—N1—Cu1	126.0 (2)	C2—C6—Cl1	111.9 (2)
C2—N1—H1	117.0	C2—C6—Cl3	106.7 (2)
Cu1—N1—H1	117.0	Cl1—C6—Cl3	110.2 (2)
N1—C2—N3	128.5 (3)	C2—C6—Cl2	112.5 (2)
N1—C2—C6	120.3 (3)	Cl1—C6—Cl2	107.50 (19)
N3—C2—C6	111.2 (3)	Cl3—C6—Cl2	108.0 (2)
C4—N3—C2	120.5 (3)	C4—C7—Cl6	107.4 (2)
N5—C4—N3	128.3 (3)	C4—C7—Cl5	110.5 (2)
N5—C4—C7	120.3 (3)	Cl6—C7—Cl5	111.2 (2)
N3—C4—C7	111.4 (3)	C4—C7—Cl4	112.4 (2)
C4—N5—Cu1	127.1 (2)	Cl6—C7—Cl4	108.0 (2)
C4—N5—H5	116.4	Cl5—C7—Cl4	107.3 (2)
			
N5—Cu1—N1—C2	−14.1 (3)	N1—C2—C6—Cl1	−140.6 (3)
N5^i^—Cu1—N1—C2	165.9 (3)	N3—C2—C6—Cl1	41.9 (4)
Cu1—N1—C2—N3	12.6 (6)	N1—C2—C6—Cl3	98.8 (3)
Cu1—N1—C2—C6	−164.4 (2)	N3—C2—C6—Cl3	−78.7 (3)
N1—C2—N3—C4	−0.4 (6)	N1—C2—C6—Cl2	−19.5 (4)
C6—C2—N3—C4	176.7 (3)	N3—C2—C6—Cl2	163.0 (3)
C2—N3—C4—N5	−5.2 (6)	N5—C4—C7—Cl6	−105.0 (3)
C2—N3—C4—C7	177.1 (3)	N3—C4—C7—Cl6	72.9 (3)
N3—C4—N5—Cu1	−2.2 (6)	N5—C4—C7—Cl5	133.5 (3)
C7—C4—N5—Cu1	175.3 (2)	N3—C4—C7—Cl5	−48.6 (4)
N1—Cu1—N5—C4	9.6 (3)	N5—C4—C7—Cl4	13.7 (4)
N1^i^—Cu1—N5—C4	−170.4 (3)	N3—C4—C7—Cl4	−168.4 (3)

Symmetry code: (i) −*x*+2, −*y*+2, −*z*.
